# Targeted proteoform degradation for precision drug design, delivery, and therapy

**DOI:** 10.1080/10717544.2026.2665871

**Published:** 2026-05-06

**Authors:** Chenyu Cui, Haoyue Zhang, Xinmiao Wang, Na Li, Xianquan Zhan

**Affiliations:** a Shandong Provincial Key Laboratory of Precision Oncology, Shandong Cancer Hospital and Institute, Shandong First Medical University & Shandong Academy of Medical Sciences, Jinan, China; b Shandong Provincial Key Medical and Health Laboratory of Ovarian Cancer Multiomics & Jinan Key Laboratory of Cancer Multiomics, Medical Science and Technology Innovation Center, Shandong First Medical University, Jinan, China; c Laboratory of Cancer Proteoformics, School of Pharmaceutic Sciences, Shandong First Medical University & Shandong Academy of Medical Sciences, Jinan, China

**Keywords:** Targeted protein degradation (TPD), targeted proteoform degradation (TPfD), proteolysis-targeting chimeras (PROTACs), proteoformics, proteoform, ubiquitin-proteasome system (UPS), E3 ubiquitin ligase, precision drug delivery, undruggable targets, personalized medicine

## Abstract

Ubiquitin-proteasome system (UPS)-mediated targeted protein degradation (TPD) is a central mechanism of maintaining protein homeostasis, which is closely associated with multiple diseases. Proteolysis-targeting chimeras (PROTACs) represent a major platform in TPD research and have demonstrated advantages relative to traditional intervention methods, such as small-molecule inhibitors, particularly in expanding the scope of targetable proteins. PROTACs have significant scientific merits in drug discovery and development. Currently, TPD still focuses on the conventional ‘protein’ level. However, the development of proteoform and proteoformics has enriched the deep understanding of the structural and functional diversities of a conventional protein. Targeted proteoform degradation (TPfD) has been proposed as an approach to more accurately recognize subtle structural and functional alterations within canonical proteins, thereby potentially improving precision drug delivery. Pathology-specific proteoform-based TPfD may facilitate the design of more precisely targetable degradation tools, potentially shifting strategies from general targeting toward more refined clearance. This review discusses the concept, mechanism, and developmental landscape of protein-based TPD and its core tool PROTACs, while systematically elaborating on the limitations of traditional TPD technologies – particularly the challenges associated with distinguishing functionally heterogeneous proteoforms. Thus, this review proposes the innovative concept of proteoform-based TPfD, detailing its technical foundations (rooted in proteoformics), core design methodologies (including ligand optimization, linker system engineering, and E3 ligase selection), and key applications in disease molecular typing, personalized drug development, and dynamic therapeutic regimen optimization. It further explores the potential role of TPfD in advancing precision medicine and discusses how proteoform-oriented strategies may contribute to refining therapeutic approaches for complex diseases.

## 1. Introduction

**Figure 1.**
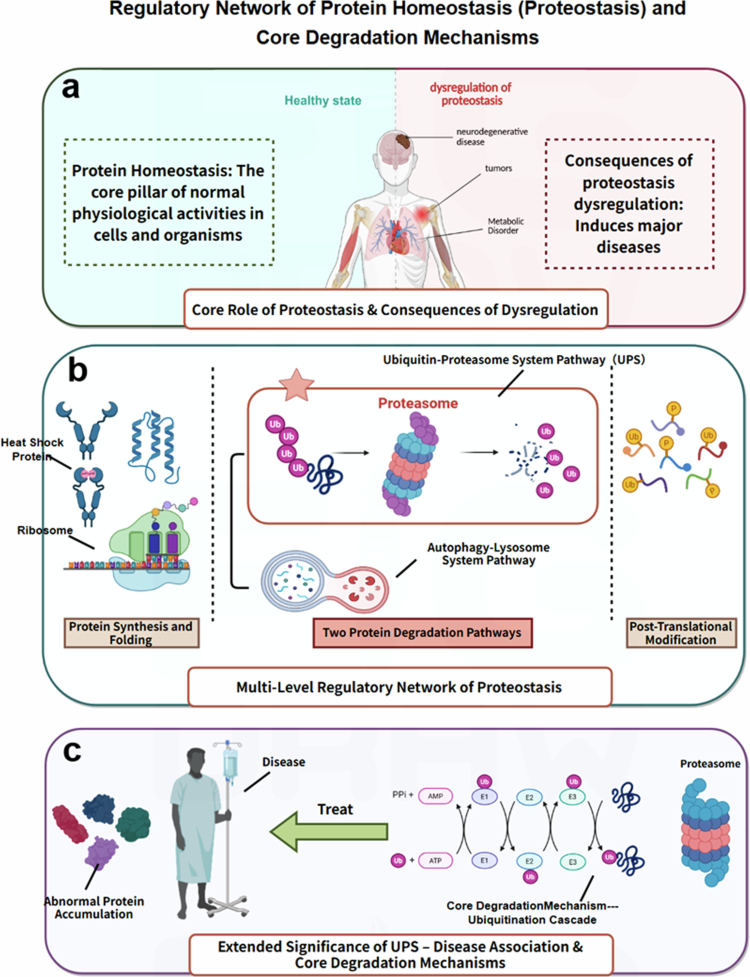
The regulatory network of protein homeostasis (proteostasis) and core degradation mechanisms. (a) The highlights that proteostasis is the core pillar of normal physiological activities in cells and organisms; its dysregulation induces major diseases. (b) The multi-level regulatory network of proteostasis, including protein synthesis and folding (mediated by ribosomes and heat shock proteins), two types of protein degradation pathways (UPS and ALP), and PTMs. (c) The extended significance of UPS; for example, UPS dysfunction leads to abnormal protein accumulation and disease, while targeted UPS modulation provides a therapeutic approach.

Protein homeostasis constitutes the core pillar of normal physiological activities in cells and organisms. Its dysregulation can trigger major diseases such as metabolic disorders, tumors, and neurodegenerative diseases (Balch et al. 2008). The maintenance of this homeostasis relies on the dynamic balance of a multi-level regulatory network. During the synthesis and folding stages, mRNAs and ribosomes play a leading role in protein synthesis; molecular chaperones such as heat shock proteins assist in correct folding; and endoplasmic reticulum quality control (ERQC) maintains balance through comprehensive monitoring of protein folding processes (Wiseman et al. 2022). The degradation mechanism serves as a crucial hub for homeostasis regulation, with two major and complementary pathways, UPS (ubiquitin-proteasome system) and ALP (autophagy-lysosome pathway), to orchestrate the intracellular protein clearance. For UPS, it labels target proteins via the E1–E2–E3 ubiquitin ligase cascade reaction (Kleiger and Mayor 2014), and the 26S proteasome complex robustly eliminates proteins with a short half-life or conformationally abnormal proteins in a highly selective, ATP-dependent manner. For ALP, it mediates the bulk or selective degradation of cytoplasmic macromolecules, misfolded proteins, and even organelles, through the formation of double-membraned autophagosomes that fuse with lysosomes for acidic hydrolysis (Chen et al. 2019). These two degradation pathways function in close coordination: UPS is primarily in charge of the rapid turnover of soluble, short-lived proteins, and ALP undertakes the clearance of insoluble protein aggregates, long-lived proteins, and subcellular structures that are inaccessible to the proteasome. Post-translational modifications (PTMs) and directed transport further participate in regulatory processes (Lee et al. 2023), and all these links form a synergistic network through protein‒protein interactions (PPI). Notably, the precise degradation mediated by UPS and ALP provides cells with a core mechanism for rapidly adjusting the proteome and resolving protein stress (Popovic et al. 2014). Dysfunction of either system can result in abnormal protein accumulation and drive disease development, which also lays a critical foundation for advancing targeted protein degradation (TPD) technologies based on both pathways (Chen et al. 2024a) (Figure 1). The ALP has emerged as a vital alternative to the UPS for TPD development, spawning novel technologies: ATTECs (autophagosome-tethering compounds), AUTACs (autophagy-targeting chimeras), and LYTACs (lysosome-targeting chimeras). These ALP-based tools address UPS limitations (e.g. poor degradation of insoluble aggregates and membrane proteins) via distinct designs: LYTACs are bifunctional molecules linking target ligands to lysosomal receptors (e.g. M6PR, ASGPR for liver targeting), which mediates endocytosis and lysosomal degradation of extracellular proteins and membrane proteins, with a representative anti-HER2 antibody-M6P conjugate efficiently degrading membrane-bound HER2, a target recalcitrant to cytosolic proteolysis-targeting chimeras (PROTACs) (Tong et al. 2023); AUTACs conjugate target ligands with guanine-derived autophagy tags, triggering TRIM21-mediated polyubiquitination and p62/SQSTM1-dependent autophagosomal degradation, and they effectively clear oncogenic Ras, mitochondrial proteins, and other soluble/membrane targets (Takahashi et al. 2019); ATTECs directly tether targets to autophagosomal membranes (via LC3 ligands) without ubiquitination/receptor recruitment, selectively degrading insoluble aggregates (e.g. mutant huntingtin in Huntington’s disease) refractory to UPS (Li et al. 2020; Xiao et al. 2025).

Nevertheless, traditional drug intervention strategies with antibody-drug conjugates (ADCs), small-molecule inhibitors, and monoclonal antibodies revolve around the clinically-validated classic mechanism called occupancy-driven mechanism (Wu et al. 2022). By virtue of their structural specificity, these agents precisely bind to and continuously occupy functional domains of target proteins (e.g. enzyme active sites, and receptor-ligand binding sites), thereby inhibiting the normal exertion of target protein functions through steric hindrance or conformational interference (Li et al. 2022). For instance, the small-molecule inhibitor A-485 binds to the catalytic active site of p300/CBP histone acetyltransferases, competitively occupying the natural binding pocket of acetyl-CoA. This blocks the transmission of the target protein's physiological functions at the molecular level, thereby inhibiting tumor proliferation and demonstrating the precision and efficacy of this mechanism (Lasko et al. 2017). In reality, however, these traditional occupancy-driven technical approaches are plagued by issues such as poor selectivity, development of drug resistance, and limited coverage of therapeutic targets beyond the scope of ligand discovery (Zhong et al. 2024), which may result in undesirable toxicity and side effects.

Inspired by the way that cells utilize protein degradation to maintain intracellular protein homeostasis, TPD technology has been developed as a revolutionary alternative to traditional occupancy-driven drugs. Different from traditional occupancy-driven drugs that passively suppress functions, TPD actively leverages the cell's natural degradation systems (e.g. UPS and ALP) to completely eliminate target proteins. Its concept is derived from the cell's inherent protein quality control mechanisms, among which PROTACs stand out as the most representative UPS-based TPD tools, while LYTACs, AUTACs, and ATTECs form the core of ALP-based TPD tools. Through artificially designed bifunctional structures of PROTACs, the natural intracellular process of recognition-labeling-degradation is mimicked, enabling the specific incorporation of pathogenic proteins into the cell’s endogenous degradative machinery and thereby accomplishing the precise clearance of target proteins (Li et al. 2020). As heterobifunctional molecules, PROTACs enlist client proteins and E3 ubiquitin ligase complexes into proximity via connecting the specific protein-targeting ligands and E3 ligase-binding ligands, thereby triggering the ubiquitination and subsequent degradation of the client protein (Guenette et al. 2022). Their recyclable event-driven mechanism allows for efficient clearance at low doses, enables the targeting of undruggable targets that are inaccessible to classical small-molecule inhibitory molecules (Samarasinghe and Crews 2021), and can significantly reduce toxicity and surmount specific drug resistance problems (Li et al. 2022). Currently, PROTAC technology has advanced rapidly, with the number of related molecules exceeding 6000, making it an important driver in the clinical translation of TPD (Tsai et al. 2024; Ge et al. 2025).

Despite their promising prospects, both UPS-based (e.g. PROTACs) and ALP-based (e.g. LYTACs, AUTACs, and ATTECs) TPD technologies face multiple challenges in practical applications. For PROTACs, the main limitations include: (i) compared to molecular glues, its larger molecular weight or higher polarity leads to poor tissue permeability (Tsai et al. 2024; Holdgate et al. 2024); (ii) the variety of available E3 ligases is limited; (iii) it is difficult to adapt to targets without well-defined catalytic pockets (e.g. most transcription factors); and (iv) the occurrence of proteoforms may lead to off-target effects (Hassan et al. 2024). For ALP-based degraders (e.g. LYTACs, AUTACs, and ATTECs), key challenges involve: (i) slow degradation kinetics and potential non-specific bulk autophagy induction leading to cytotoxicity (Qian et al., 2021); (ii) limited tissue and cell specificity of lysosomal receptor ligands (e.g. M6PR ligands show poor tumor selectivity) (Berger et al., 2020); (iii) the instability of synthetic conjugates in the extracellular environment; and (iv) the same inability to distinguish functionally heterogeneous proteoforms such as PROTACs, which is a common core bottleneck for all traditional TPD technologies.

Among these challenges, the one most directly related to precise disease treatment and the primary focus of this article is the inability of all current TPD tools (PROTACs, LYTACs, AUTACs, and ATTECs) to accurately recognize proteoforms. A canonical protein can contain a set of proteoforms derived from one gene due to PTMs, alternative splicing, or genetic variations, and these isoforms make distinct contributions to disease development (Noor et al. 2021). Traditional TPD technologies are generally designed at the canonical protein level, which do not fully account for proteoform heterogeneity. This limitation constrains their precision and therapeutic efficacy, which, in turn, highlights the potential value of proteoformics in advancing personalized drug therapy (Cui et al. 2020).

As an emerging research field focused on proteoforms, proteoformics is dedicated to systematically decipher the sources of proteoform diversity (e.g. genetic variations, alternative splicing, PTMs, spatial conformation, etc.), their dynamic variation patterns, and their associations with physiological functions and pathological processes. It has been acknowledged that a single canonical protein can exist in different proteoforms, and the between-proteoform distinctions may contribute directly to variations in drug responses (Su et al. 2024). By employing techniques such as mass spectrometry (particularly the top–down approach, which directly analyzes intact proteoforms and overcomes the bottleneck of assigning peptide information to original proteoforms in bottom-up approaches) (Kaulich and Tholey 2025) and single-cell proteomics, proteoformics provides analytical tools that facilitate the identification of disease-specific pathogenic proteoforms. It may offer potential molecular targets and a theoretical framework for developing personalized drugs that precisely target specific pathogenic proteoforms, thereby supporting the shift toward more refined and precise targeting strategies. It is against this backdrop that the concept of targeted proteoform degradation (TPfD) has emerged. Unlike TPD that targets the canonical protein, TPfD shifts its focus from the canonical protein to specific proteoform subtypes. By leveraging the precise analysis of targeted proteins with proteoformics, TPfD is proposed as a strategy that may facilitate the design of degradation tools capable of adapting to target proteoforms. Such an approach may help reduce unintended degradation of non-pathogenic variants and potentially mitigate certain forms of drug resistance. This study opens up a new path for precise drug design, delivery, and therapy: allowing drugs to act exclusively on key disease-driving factors with the potential to improve therapeutic outcomes while reducing adverse effects. Recent studies have provided experimental evidence for the feasibility of proteoform-selective degradation. For instance, a biological PROTAC (BioPROTAC) was developed to selectively degrade misfolded SOD1 while sparing wild-type SOD1 in models of amyotrophic lateral sclerosis (Chisholm et al. 2025). Similarly, a SPOP-based BioPROTAC was engineered to specifically target the NUP98:KDM5A fusion oncoprotein in acute myeloid leukemia without affecting wild-type NUP98 (Kirkiz et al. 2025). In the context of small molecules, the first PROTAC was reported to be capable of degrading endogenous KRASG12C mutant while sparing other KRAS variants (Bond et al. 2020). These proof-of-concept studies demonstrate that TPfD is not merely a theoretical concept but an achievable strategy with demonstrated experimental validation.

## 2. Characteristics of TPD vs. small-molecule occupancy-driven drug strategy

### 2.1. Small-molecule occupancy-driven drug strategy

The development of conventional pharmaceuticals has long centered on the direct modulation of protein activity, with inhibitors representing one of the most extensively utilized categories. These small-molecule drugs mainly inhibit the functions of target proteins by occupying their active sites or binding sites, such as enzyme activity, receptor signal transduction, etc. This occupancy-driven mechanism requires small molecules to bind tightly to the functional sites of target proteins, such as catalytic pockets or allosteric sites, thus blocking their activities, and it only acts on proteins with clearly defined binding pockets (approximately 20% of the human proteome), and it cannot eliminate the non-enzymatic functions of the proteins (Zhou et al. 2023; Campos et al. 2025). For example, kinase inhibitors prevent the binding of substrates or cofactors by competitively binding to the active sites (Riha et al. 2024). Agonists or antagonists activate or inhibit signaling pathways by regulating the conformational changes of receptor proteins (Qin et al. 2022).

The limitations of conventional small-molecule occupancy-driven drugs (e.g. small-molecule inhibitors) include (i) the strong target dependence: only applicable to proteins with well-defined binding pockets (approximately 20% of the human proteome) and ineffective against undruggable targets lacking such structures (Duran-Frigola et al. 2023; Campos et al. 2025); and (ii) drug resistance: long-term use may lead to mutations within the binding domain of the target proteins or the activation of compensatory pathways, reducing therapeutic efficacy (Kim et al. 2022; Lee et al. 2025). Therefore, conventional small-molecule occupancy-driven targeted therapy has obvious disadvantages in the field of drug therapy.

### 2.2. TPD

TPD is an event-driven mechanism of action, which needs to design the corresponding degraders, such as PROTACs to initiate the TPD process for targeted therapy, which is much better than small-molecule occupancy-driven drug therapy. However, the design of TPD degraders is focus on the protein level, which cannot distinguish different proteoforms that are derived from a canonical protein, potentially preferentially degrading non-pathogenic subtypes and resulting in the expansion of drug-resistant clones (Wang et al. 2024). Additionally, they cannot completely inhibit the function, only inhibits the activity of proteins, but fails to eliminate their non-enzymatic functions or scaffolding roles (VanDyke et al. 2022; Guedeney et al. 2023). Simultaneously, due to the inability to recognize conformational differences caused by PTMs or mutations, the degradation efficiency of specific proteoforms with abnormal functions may be insufficient (Münick et al. 2024). Finally, it is unable to address the functional heterogeneity of proteoforms. For example, the dimer (promotes tumor proliferation) and tetramer (maintains basic metabolism) of PKM2 exhibit distinctly different functions; however, those two subtypes of PKM2 cannot be selectively degraded with traditional TPD, which may fail to block tumor metabolic reprogramming and may even retain pathogenic subtypes (Münick et al. 2024). This clearly demonstrated that TPD has obvious limitations in discriminating the functional heterogeneity of PKM2 subtypes. Therefore, novel degraders are developed for improving TPD efficiency.

## 3. Development of TPD degraders and the importance of PROTACs

The development of TPD technology has been marked by innovations and breakthroughs, with its core trajectory centered on the development and expansion of protein degradation systems.

In the late 1990s, the concept of TPD was formally proposed, inspired by observations of the mechanisms by which viruses and plants regulate protein levels. Notable examples include the human papillomavirus (HPV) E6 protein, which recruits the E3 ubiquitin ligase E6AP to promote the p53 degradation (Talis et al. 1998), and the plant hormone auxin (IAA), which can regulate protein degradation by stabilizing the interaction between the Aux/IAA transcriptional repressor and the E3 ligase Tir1 (Gray et al. 2001). These phenomena provided natural templates for the artificial design of degradation tools.

In 2001, the first proof-of-concept peptide-based PROTAC was synthesized. It successfully degraded methionine aminopeptidase 2 (METAP2), demonstrating for the first time that the UPS could be artificially manipulated. This milestone marked the entry of TPD technology into a phase of substantial development (Sakamoto et al. 2001). Built on the pioneering application of the UPS system, PROTACs accomplish the precise recruitment of client proteins and E3 ligases through multi-specific design and the induced-proximity mechanism, representing a landmark achievement of the UPS system in the field of TPD.

Subsequently, researchers continuously optimized the PROTAC design. For instance, the development of designed ankyrin repeat proteins (DARPins) significantly accelerated the development efficiency of bioPROTACs and other types (Münick et al. 2024). Meanwhile, molecular glues (MGs), another class of UPS-dependent systems, have gradually emerged. Compounds such as thalidomide and its analogs trigger the ubiquitination and degradation of client proteins by enhancing PPIs, together with PROTACs, enriching the toolkit of UPS-mediated TPD (Oleinikovas et al. 2024).

With the deepening of technical exploration, proteasome-independent degradation systems (e.g. endosomes, lysosomes, and autophagosomes) have also become new directions for development, which has led to the creation of novel technologies such as chimeric molecules targeting lysosomes (LYTACs), chimeric constructs directed at autophagy (AUTACs), and compounds that tether to autophagosomes (ATTECs), further expanding the application boundaries of TPD (Takahashi et al. 2019; Zhong et al. 2025; Shao et al. 2025). Strategies to improve the pharmacokinetic properties of PROTACs are urgently needed. One promising approach is the conjugation of degraders to long-circulating carriers such as the Fc domain of antibodies. A study demonstrated that Fc-conjugation significantly increased the half-life of the SMDC EC140 from half an hour to 28 hours while maintaining target specificity and enhancing antitumor efficacy (Zheng et al. 2025). This Fc-based delivery strategy could be adapted for TPD degraders such as PROTACs to improve their circulation time, tissue penetration, and overall therapeutic index. Future development of Fc-conjugated PROTACs or nanoformulations may help overcome the pharmacokinetic barriers that currently limit their clinical translation.

Notably, PROTACs have particularly remarkable clinical translation progress. In 2019, ARV-110 (directed against the androgen receptor) and ARV-471 (specific for the estrogen receptor) initiated phase I clinical studies, verifying the feasibility of PROTACs in humans for the first time and marking an important step toward a clinical translation agent (Békés et al. 2022). According to statistics from the open-access database PROTAC-DB, a total of 6111 PROTAC molecules have been developed within just a few decades (Ge et al. 2025). This explosive growth not only confirms the maturity of the technology but also indicates that, in the short term, most TPD therapeutics entering clinical development will still be centered on PROTACs (Tsai et al. 2024). PROTACs currently occupy a central and influential position in the evolution of TPD technology.

## 4. Mechanism of PROTACs

The PROTAC-based TPD is an event-driven mechanism of action. Briefly, the degraders PROTACs recruit E3 ligases to the target protein, prompting its ubiquitination and subsequent proteasomal destruction. A single degrader can cyclically degrade multiple rounds of target proteins, offering advantages such as low dosage and long-lasting effects. Moreover, it can overcome certain undruggable targets, such as transcriptional factors without active sites (Yarawsky et al. 2025; Krone and Crews 2025). Thereby, PROTAC-based TPD can overcome the target binding limitations of small-molecule occupancy-driven drugs.

**Figure 2.**
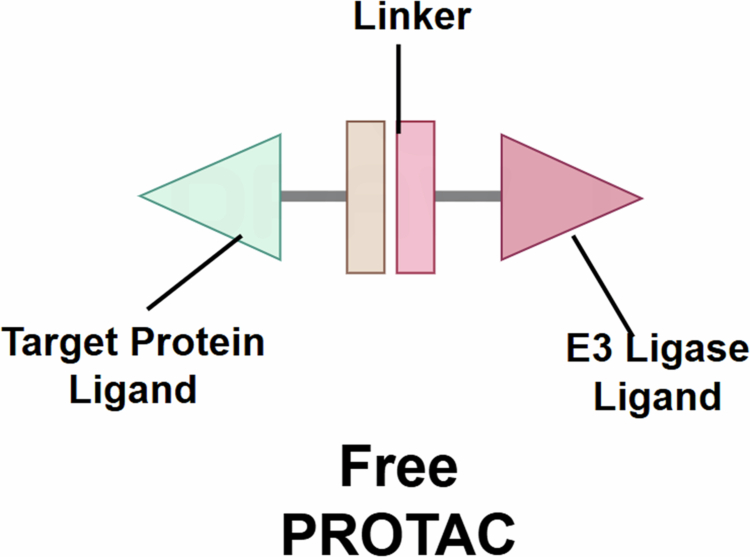
Structure of free PROTAC. The target protein ligand is designed to accurately recognize the target protein, and the E3 ligase ligand is designed to ubiquitinate a target protein. Linker is designed to link E3 ligase and target protein ligands, and regulate the activities and selectivity of PROTAC through the length and chemical properties of the linker. The term ‘free PROTAC’ refers to the unbound state of the molecule prior to engaging either the target protein or the E3 ligase. This state is critical for cellular permeability and initial diffusion. Upon target engagement, the PROTAC forms binary and ternary complexes (Figure 3), which are essential for inducing ubiquitination and degradation. Understanding the structure of the free PROTAC is fundamental to optimizing its pharmacokinetics and degradation efficiency.

PROTACs represent a novel therapeutic strategy for achieving TPD via the UPS (Xie et al. 2023; Song et al. 2023). Their mechanism hinges on the design of bifunctional molecules, which induce the proximity of the target protein and E3 ubiquitin ligase, then the target proteins are ubiquitinated, followed by degradation by the proteasome. This strategy has transformed the therapeutic targeting of proteins historically deemed undruggable by conventional small-molecule inhibitors. Various drug discovery campaigns have foundered due to insufficient selectivity or inadequate tissue distribution of inhibitory compounds. PROTACs and MGs address these limitations by enabling spatiotemporally controlled target inactivation through induced proximity. Structurally a typical PROTAC comprises three key elements: (i) Target protein ligand, which specifically binds to the protein of interest, ensures the PROTAC accurately recognizes the target protein; (ii) E3 ligase ligand, which recruits a specific E3 ubiquitin ligase, provides a catalytic platform for the ubiquitination of the target protein; and (iii) Linker, which serves as the critical bridge between E3 ligase ligand and target protein ligand, exerts a crucial impact on the activity and selectivity of the PROTAC through its length and chemical properties (Chen et al. 2022) (Figure 2).

**Figure 3.**
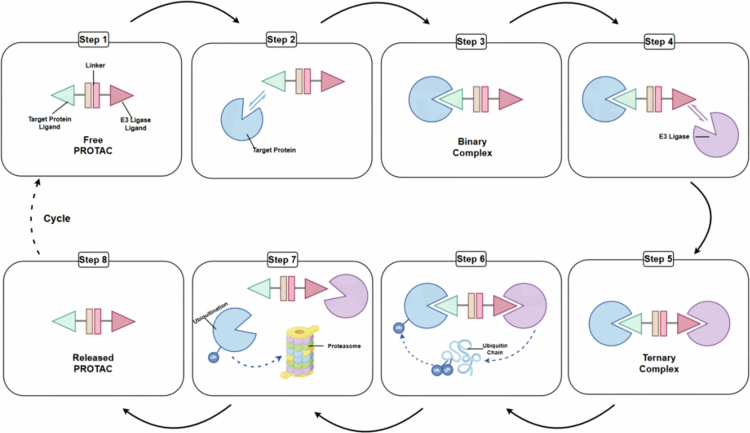
PROTAC-mediated protein degradation mechanism. This figure illustrates the cyclic process of PROTAC-driven degradation of the target protein. First, a PROTAC is bound to a target protein to generate a binary complex, then an E3 ligase is recruited, which finally forms a ternary complex. Then, ubiquitin chains, which are impacted by E3 ligase are transferred to the target protein; the 26S proteasome identifies and processes the ubiquitin-tagged target protein for destruction, while the PROTAC is released and recycled to initiate new rounds of degradation.

The PROTAC mechanism operates as follows: First, targeted protein recognition. The PROTAC molecule engages the target protein via its target protein ligand, forming a PROTAC-target protein complex (VanDyke et al. 2022). Second, E3 ligase recruitment. The PROTAC molecule subsequently recruits its cognate E3 ubiquitin ligase. This recruitment culminates in the assembly of a ternary complex, a catalytically active assembly essential for the ubiquitination process (PROTAC-target protein-E3 ligase) (Xiong et al. 2022). Third, ubiquitination and degradation. Finally, ubiquitination and degradation are accomplished. The E3 ligase catalyzes ubiquitin molecules to be transferred onto target protein, initiating a ubiquitination cascade. This ubiquitin tag is a molecular signal for subsequent recognition and ultimate degradation of the target protein by proteasome (Chen et al. 2022) (Figure 3).

PROTAC exhibits multiple advantages and innovations. In terms of enhanced selectivity, by optimizing the length of the linker and the stability of the ternary complex, PROTAC can achieve highly selective degradation of target proteins, even surpassing the selectivity of its parent ligands (Chen et al. 2022). To broaden the range of targetable proteins, PROTAC technology enables the targeting of proteins intractable to conventional small-molecule inhibitors, such as proteins lacking catalytic activity or binding sites (Xiong et al. 2022). Meanwhile, it also possesses tissue specificity; by leveraging the tissue distribution characteristics of E3 ubiquitin ligases, PROTAC can realize spatially controlled degradation of protein targets or cell types (Chen et al. 2022). There are various innovative variants of PROTAC. Bridged PROTAC recruits E3 ubiquitin ligase through the binding partner of the target protein-binding ligand, enabling the disposal of target proteins (e.g. cyclin D1) that lack small-molecule ligands (Xiong et al. 2022); dual-acting PROTAC (DAO-PROTAC) is only activated when two disease-related endogenous stimuli are present simultaneously, thereby reducing off-target toxicity (Dutta et al. 2025); and bioPROTAC refers to PROTACs constructed using biomolecules such as single-domain antibodies (VHH) and TRIM21, which can degrade difficult-to-target proteins (e.g. HuR) (Fletcher et al. 2023). PROTAC holds great potential in clinical applications. In cancer therapy, it has been successfully used to degrade a variety of cancer-related proteins, such as KRAS mutants, NR4A1, and MDM2, demonstrating significant anti-tumor effects (Adams et al. 2023; Wang et al. 2024). In the field of aging-related diseases, by targeting signaling pathways shared by cancer and aging, PROTAC provides a novel idea in the clinical management of conditions linked to aging (Peng et al. 2024). In immunotherapy, through degrading immunosuppressive proteins (e.g. NR4A1), PROTAC can enhance anti-tumor immune responses (Wang et al. 2024).

PROTACs possess distinct advantages relative to conventional small-molecule inhibitors, addressing the key limitations of traditional inhibitory strategies. PROTACs catalytically mediate multiple rounds of degradation of target protein in a sub-stoichiometric way, in contrast to small-molecule inhibitor drugs that stoichiometrically occupy the protein active sites to only modulate the function of the target protein, and PROTACs achieve far more potent degradation efficiency with nanomolar DC50 values and over 90% degradation rate. PROTACs induce remarkable target protein depletion within 1 or 2 h after treatment, and the degradation effect can be sustained for 24 or even 48 h, even after the removal of PROTACs, while the inhibitory effect of small-molecule inhibitors relies on sustained drug concentrations and recovers rapidly after drug withdrawal. PROTACs can rapidly, sustained, and robustly inhibit downstream signaling pathways through completely degrading target protein, which avoids the incomplete inhibition of downstream signals caused by compensatory accumulation of target proteins induced by small-molecule inhibitor drugs, and also abrogates the scaffolding roles of multidomain proteins to prevent kinome rewiring and compensatory feedback activates downstream signaling cascades triggered by small-molecule inhibitor drugs that only disrupt one domain activity of such a protein. In tumor xenografts, PROTACs can exert stronger suppression roles of in vitro cell proliferation and induce apoptotic cell death, more profound in vivo tumor growth inhibition, and significantly improve the survival rate of model mice compared to small-molecule inhibitors. PROTACs can overcome mutation-derived drug resistance, as small-molecule inhibitors tend to lose binding capacity because of nonsynonymous mutations at the protein active sites, whereas PROTACs only transiently and reversibly associate with target proteins to mediate degradation, thus effectively degrading mutant target proteins such as BTK C481S and EGFR with exon 20 insertion. PROTACs display enhanced target selectivity that surpasses the intrinsic binding-specificity of target protein ligand (e.g. JQ1 does not have selectivity for BRD2/3/4 isoforms, but JQ1-based PROTACs can induce selective depletion of BRD4), and proteome-wide approaches have verified that PROTACs only degrade a few target proteins, resulting in much lower off-target effects than small-molecule inhibitors (An and Fu 2018).

## 5. Limitations of PROTAC-based TPD

Although PROTAC technology shows great potential in the field of drug development, it still faces a full chain of challenges ranging from molecular design and mechanism of action to target specificity in practical applications, which significantly restricts the exertion of its precise therapeutic efficacy. Also, PROTAC-based TPD fails to address the issue of functional heterogeneity of proteoforms, which has become a core bottleneck restricting further improvements in its precision.

In terms of molecular properties, PROTAC is formed by the covalent linkage of a client protein ligand and an E3 ligase ligand to recognize ubiquitin via a linking unit, with a molecular weight usually ranging from 1000 to 2000 Da. Its large molecular size and exposed polar surface area make it difficult to penetrate cell membranes and tissue barriers, directly affecting delivery efficiency (Cecchini et al. 2021; Tsai et al. 2024; Holdgate et al. 2024). Although strategies for improvement – such as controlling the molecular weight below 1000 Da and using liposomes or nanoparticles for delivery –have been explored, the effectiveness of these methods still needs long-term verification in combination with specific targets and disease scenarios, and the optimization process remains long and arduous (Klein et al. 2020). Recent efforts in specific cancer contexts, such as ovarian cancer, have highlighted the importance of formulation strategies and delivery systems to boost PROTAC bioavailability, underscoring the need for cancer type-specific optimization (Rejili et al. 2025). Strategies to improve the pharmacokinetic properties of PROTACs are urgently needed. One promising approach is the conjugation of degraders to long-circulating carriers such as the Fc domain of antibodies. A study found that Fc-conjugation significantly increased the half-life of the SMDC EC140 from half an hour to 28 h while maintaining target specificity and enhancing antitumor efficacy (Zheng et al. 2025). This Fc-based delivery strategy could be adapted for PROTACs or TPD molecules to improve their circulation time, tissue penetration, and overall therapeutic index. Future development of Fc-conjugated PROTACs or nanoformulations may help overcome the pharmacokinetic barriers that currently limit their clinical translation. In addition to the aforementioned delivery challenges, the development of advanced nanomedicines offers promising strategies to improve targeted drug delivery and modulate local pathology. For instance, ADCs are a category of targeted therapeutics that integrate the specific monoclonal antibodies and potent cytotoxic payloads, although they face resistance mechanisms such as impaired internalization, antigen loss, and drug efflux (Eltaib et al. 2025). Clarification of these drug-resistance mechanisms is essential for designing next‑generation targeted degraders. Furthermore, multifunctional nanoplatforms have been developed that integrate targeting, therapy, and modulation of the local microenvironment. A study reported a tea polyphenol‑derived nanomedicine functionalized with cRGD for targeted photothermal thrombolysis, which simultaneously exhibits free radical scavenging and anti‑inflammatory activities, thereby addressing both thrombus removal and inflammation suppression (Wang et al. 2024). Such strategies highlight the potential of combining therapeutic and microenvironment‑modulating functions within a single nanocarrier, which could be adapted for TPfD to enhance efficacy and reduce side effects. These technological advances collectively contribute to the realization of precision medicine.

In the core link of the mechanism of action, there are obvious limitations in the selection and utilization of E3 ubiquitin ligases. Currently, only a few types of E3 ligases (e.g. VHL and CRBN) dominate in TPD technology (Dale et al. 2021; Weng et al. 2021). This reliance on a few and repeated use models may lead to drug resistance, as well as uneven degradation efficiency caused by differences in tissue/cell specificity (Toma-Fukai and Shimizu 2021).

The challenges in target adaptation and specificity are even more complex. On the one hand, most PROTAC targets still follow the known targets of traditional drugs. In clinical practice, there is a need to verify these agents’ unique advantages over traditional therapies (e.g. small-molecule inhibitors and antibodies), such as more thorough intervention achieved by degradation rather than inhibition, and this verification process is often time-consuming and costly (Békés et al. 2022). On the other hand, TPD faces difficulties in ligand screening: approximately 80% of human proteins lack small-molecule ligands, which limits the scope of targets. For proteins without clear catalytic pockets (e.g. most transcription factors), it is difficult to design high-affinity ligands (Nalawansha and Crews 2020), and only a few specific proteins can be effectively degraded (Samarasinghe and Crews 2021).

In the advancement of PROTAC technology, the complexity introduced by proteoforms has attracted increasing attention, as it may influence degradation selectivity and therapeutic precision. A fundamental question arises: Can current PROTACs or other degraders discriminate among distinct proteoforms that are derived from a single canonical protein? To date, the answer is largely negative. Glial fibrillary acidic protein (GFAP) is taken as an example, which is affected by PTMs (e.g. phosphorylation, glycosylation, acetylation, ubiquitination, and nitration) to form multiple proteoforms with different structures and functions. As a biomarker for reactive astrogliosis in the central nervous system, GFAP undergoes changes in levels after brain injury and neurological diseases, and its proteoform diversity may complicate detection and clinical interpretation (Gogishvili et al. 2025). Similarly, proteoform diversity in other proteins may add an additional layer of complexity to precise degradation strategies.

While several isoform-selective PROTACs have been successfully reported (Maurice 2025; Haag et al. 2025), systematic discrimination at the proteoform level – particularly those arising from PTMs – remains relatively underexplored. Consequently, degraders designed based on canonical protein sequences may potentially result in non-selective degradation among closely related variants.

To date, direct clinical evidence demonstrating that proteoform heterogeneity has caused documented PROTAC failure remains limited. Nevertheless, as precision medicine continues to evolve, achieving higher-resolution target recognition at the proteoform level may represent an important direction for future development.

## 6. Functional heterogeneity of proteoforms and the necessity of targeting

In 2012, a new nomenclature ‘proteoform’ was proposed to represent different, distinct protein molecular forms derived from a single gene, mainly focusing on two important dimensions – the amino acid sequence and PTMs of a proteoform (Smith and Kelleher 2013). Further, the complete definition has been clarified as the integration of amino acid sequence, PTMs, spatial conformation, binding partners, cofactors, localization, and biological functions (Zhan et al. 2019; Zhan 2020; Zhan et al. 2018a, 2018b), referring to the final structural and functional states of a protein, which is the basic functional unit of the proteome.

The concept of protein molecular diversity has been explored previously with another term, ‘protein species’ (Jungblut et al. 2008; Schlüter et al. 2009), while ‘moonlighting proteins’ describes a different phenomenon where a single protein performs multiple functions without structural changes (Jeffery 1999; Wu et al. 2025). The protein morphological diversity is mainly due to the influence of multiple parameters, including genomic variations, alternative splicing, post-transcriptional modification, and PTMs (Zhan et al. 2018, 2019; Riha et al. 2024; Campos et al. 2025). For example, genomic variations such as mitochondrial single-nucleotide polymorphisms (mSNPs) can result in different proteoforms. Also, RNA splicing and modifications, protein PTMs, and proteolytic processing can contribute to the diversity of a protein. The development of two-dimensional gel electrophoresis in combination with mass spectrometry (2DE-MS) and top-down MS (TD-MS) enables the identification and characterization of protein morphological diversity (Zhan et al. 2018b; Li et al. 2021).

**Figure 4.**
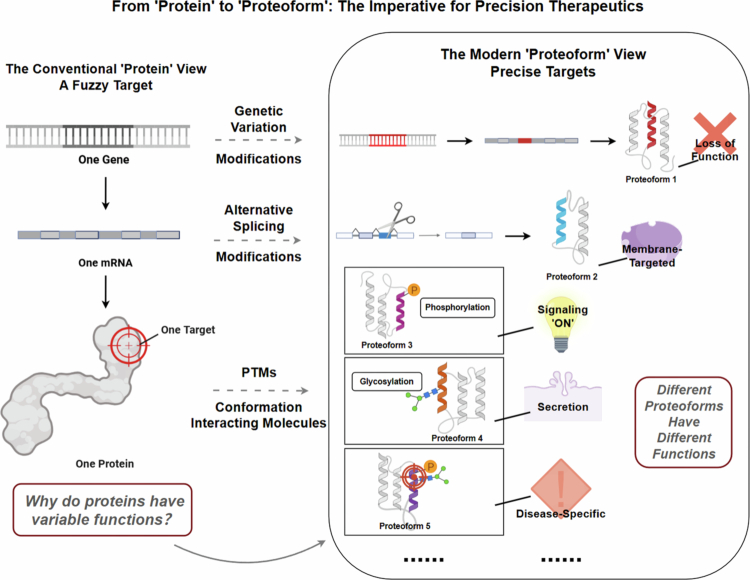
From ‘protein’ to ‘proteoform’: The imperative for precision therapeutics. Left Panel (The conventional protein view): This panel illustrates the historical ‘one gene → one protein → one target’ paradigm. This model, which conceptualizes a protein as a single, monolithic entity, results in imprecise and variable therapeutic outcomes. Right Panel (The modern proteoform view): In contrast, this panel depicts the contemporary framework based on proteoforms. It demonstrates how a single genetic locus can give rise to a spectrum of structurally and stoichiometrically distinct proteoforms via mechanisms, including genetic variation, alternative splicing, and PTMs such as phosphorylation and glycosylation. These distinct proteoforms mediate unique and often discrete biological functions (e.g. signal activation, membrane localization, secretion, or loss of function). Crucially, specific, disease-associated proteoforms (e.g. proteoform 5) emerge as targets for highly specific therapeutic interventions, thereby promising enhanced efficacy and mitigated off-target effects.

In 2023, another new nomenclature ‘proteoformics’ was coined to represent the theories and methodology system for studying proteoforms – the morphological diversity of a proteome (Zhan et al. 2023; Yang et al. 2023; Su et al. 2024; Fang et al. 2025). Here clearly demonstrates that proteoform is much different from the canonical protein (Zhan et al. 2019; Su et al. 2024; Fang et al. 2025; Yang et al. 2025) (Figure 4).

### 6.1. Functional heterogeneity

PTMs, post-transcriptional regulations, and genomic variations are the common factors to cause the morphological diversities of proteins (Zhan et al. 2018a, 2019; Palacios 2022; Yarawsky et al. 2025), which present distinct proteoforms from a single gene, and such variants often exhibit functionally heterogeneous properties that directly impact physiological homeostasis and disease progression.

#### 6.1.1. Genomic variations

Single nucleotide polymorphisms (SNPs) or mutations in genomic sequences are key drivers of proteoform diversity. For instance, in chronic lymphocytic leukemia (CLL), somatic mutations in the splicing factor gene SF3B1 (e.g. K700E) alter its amino acid sequence, leading to the production of mutant SF3B1 proteoforms (Rossi et al. 2011). Unlike wild-type SF3B1, which mediates accurate pre-mRNA splicing, these mutant proteoforms aberrantly regulate the splicing of downstream target genes (e.g. BCL2L11), promoting cancer cell survival and drug resistance. Another example is the oncogenic KRAS protein: genomic variations such as G12D or G12V mutations generate KRAS proteoforms with constitutive GTPase activity, which constitutively activate the MAPK signaling pathway to drive tumor proliferation, whereas wild-type KRAS exhibits tightly regulated GTP/GDP cycling (Popow et al. 2024). These cases illustrate how genomic variations directly reshape proteoform function to contribute to disease pathogenesis.

#### 6.1.2. Post-transcriptional regulation

Alternative splicing, a major post-transcriptional regulatory mechanism, generates proteoforms with distinct domain compositions and biological functions. In pituitary neuroendocrine tumors, the growth hormone (GH) gene undergoes tissue-specific alternative splicing, producing multiple GH proteoforms. For example, the full-length GH proteoform (GH1-217) exerts classic somatotrophic effects by binding to the GH receptor (GHR) in liver and muscle tissues, while a truncated splice variant (GH1-176) lacks the GHR-binding domain and instead regulates immune cell function via non-canonical signaling pathways. Similarly, in breast cancer, alternative splicing of the CD44 gene generates standard (CD44s) and variant (CD44v) proteoforms: CD44s maintains epithelial cell homeostasis, while CD44v6 (a variant with additional exons) interacts with c-Met to promote cancer stem cell self-renewal and metastasis (Campos These examples demonstrate that alternative splicing expands proteoform functional diversity by modifying protein structure and interaction networks.

#### 6.1.3. PTMs

PTMs such as phosphorylation, glycosylation, and ubiquitination dynamically regulate proteoform function by altering their conformational states, subcellular localization, or binding partners. For example, the protein tau in Alzheimer’s disease (AD) undergoes increased phosphorylation levels at serine/threonine residues (e.g. Ser396, and Thr231), generating pathogenic tau proteoforms that lose microtubule-stabilizing activity and aggregate into neurofibrillary tangles – key pathological hallmarks of AD (Parra Bravo et al. 2024). In contrast, physiological low-level phosphorylation of tau is essential for maintaining microtubule dynamics in neurons. Another example is cardiac troponin I (cTnI) in hypertrophic cardiomyopathy (HCM). Specific phosphorylation of cTnI at Ser23/24 by protein kinase A generates a pathogenic proteoform that disrupts the troponin-tropomyosin complex, impairing myocardial contractility and leading to cardiac hypertrophy (Tucholski et al. 2020). Additionally, in sigmoid colon cancer, abnormal ubiquitination of target proteoforms (regulated by deubiquitinases such as USP10) prevents their degradation, resulting in the accumulation of oncogenic proteins that drive tumor proliferation and metastasis (Yang et al. 2023). These studies confirm that PTMs are critical for switching proteoform functions between physiological and pathological states.

Different proteoforms derived from the same gene thus exhibit distinct biological roles, and their dysregulation is closely linked to disease development. Studies in personalized drug therapy have further indicated that such functional heterogeneity directly affects drug responses: for example, mutant KRAS proteoforms (G12D/V) are resistant to traditional small-molecule inhibitors but can be selectively degraded by PROTAC-like molecules (e.g. ACBI3), while wild-type KRAS remains unaffected (Popow et al. 2024; Su et al. 2024). This functional divergence highlights the necessity of targeting specific proteoforms for precise therapeutic intervention.

### 6.2. Necessity of targeting

When canonical proteins are used as targets for personalized drugs, obvious drawbacks emerge, as a single spliced protein may exist in multiple proteoforms. These proteoforms can undergo changes such as modifications and binding in cells, tissues, or diseases, which result in differences in functions and activities to affect the accuracy of personalized drugs to interact with their target proteins. For example, in drug design and personalized therapy, when a drug is developed to target a canonical protein that has multiple proteoforms, the drug might be effective against some proteoforms but ineffective against the others, resulting in adverse effects or failure to achieve the expected therapeutic outcomes (Su et al. 2024). Understanding the complex network of protein-protein interactions (PPIs) and protein complexes is essential for deciphering the functional context of proteoforms and designing precise targeting strategies. Computational approaches have emerged as powerful tools with the use of PPI networks to systematically identify protein complexes (Meng et al. 2022). For instance, the DPCMNE framework is developed to employ a multi-level network system to attain global and local topological information in biological networks (Meng et al. 2022). This method enables the detection of protein complexes with higher biological significance by integrating hierarchical network compression with core-attachment-based strategies (Meng et al. 2022). Such computational tools provide a network-level understanding of how proteoforms assemble into functional complexes and how disease-associated variants may perturb these interactions. Integrating these approaches with proteoformics could facilitate the identification of proteoform-specific interaction networks and guide the design of TPfD molecules that target pathogenic proteoforms within their native complex contexts (Meng et al. 2022; Fang et al. 2025).

To achieve personalized drug therapy that better aligns with the specific needs of patients and disease characteristics, there is a need to shift drug targeting from canonical proteins to proteoforms. Proteoformics research can in-depth clarify disease mechanisms, and identify new signaling pathways and drug targets, offering the scientific data and theoretical basis to develop novel drugs. It can also help physicians to assess health risks and formulate more cost-effective strategies for targeted prevention (Su et al. 2024; Fang et al. 2025). Therefore, targeted therapy against proteoforms is crucial for improving drug efficacy, reducing side effects, and advancing various aspects of personalized drug therapy (Table 1).

**Table 1.** Comparison of targeting strategies for protein or proteoform intervention.

Targeting strategy	Ability to recognize proteoforms	Mechanistic characteristics	Examples	Potential issues	References
Small-molecule occupancy-driven drug strategy (e.g. small-molecule inhibitors	Unable to distinguish proteoforms.	Binds to and blocks the active site to inhibit function.	Kinase inhibitors (e.g. imatinib) for chronic myeloid leukemia. Mutations in the BCR-ABL kinase domain (e.g. T315I) lead to loss of inhibitor binding and drug resistance.	1. Only inhibits partial subtypes of the target; non-pathogenic proteoforms with the same binding domain remain unaffected. 2. Cannot block non-enzymatic functions (e.g. scaffolding roles) of target proteins. 3. High risk of drug resistance due to target protein mutations in binding domains.	Lasko et al. (2017); Li et al. (2022); Wu et al. (2022); Kim et al. (2022); Lee et al. (2025)
TPD	Non-selective degradation of multiple proteoforms derived from the same gene; lacks specificity for proteoform-specific structural features (e.g. unique PTMs, amino acid mutations)	Recruits E3 ligases to induce ubiquitination and proteasomal degradation of the target protein. A single degrader can act catalytically.	Degradation of total PRMT5 or MDM2 protein levels, without differentiating between their functionally distinct splice variants or PTM states.	1. May degrade non-pathogenic proteoforms, disrupting normal physiological functions. 2. Pathogenic proteoforms with altered degradation-related epitopes may escape degradation, leading to clonal expansion. 3. Poor tissue permeability due to large molecular weight.	Kim et al. (2022); Békés et al. (2022); Adams et al. (2023); Tsai et al. (2024); Samarasinghe et al. (2021)
TPfD	Specifically recognizes functionally abnormal proteoforms via proteoform-specific epitopes (e.g. mutation-induced conformational changes, disease-specific PTMs)	Integrates proteoformics analysis to design degraders that selectively bind and degrade only the disease-driving proteoform.	Selectively degrading misfolded SOD1. (e.g. SOD1-BioPROTAC). Targeting the NUP98:KDM5A fusion protein; and SPOP-based BioPROTAC).	1. High technical difficulty in designing proteoform-specific ligands, requiring advanced structural biology and screening. 2. Dependence on tissue-specific E3 ligases may limit application in certain organs.	Zhan et al. (2019); Su et al. (2024); Chisholm et al. (2025); Kirkiz et al. (2025); Fang et al. (2025)

Note: This table systematically compares three core targeting strategies for protein or proteoform intervention (small-molecule occupancy-driven drug strategy, TPD, and TPfD) from six dimensions: proteoform recognition ability, mechanistic characteristics, potential limitations, representative examples, and therapeutic efficacy impact. This highlights the evolutionary progression from ‘non-specific inhibition/degradation’ to ‘precision proteoform targeting,’ clarifying the technical advantages of TPfD in avoiding off-target effects, reducing drug resistance, and improving therapeutic safety – providing a theoretical basis for the application of TPfD in precision medicine. Examples cited are derived from the research content of the manuscript, ensuring consistency with the study’s technical context and practical implications.

## 7. Technical foundation and application value of proteoformics for TPfD

Proteoformics utilizes high-throughput technologies to systematically characterize the composition, dynamics, and functional implications of proteoforms. Key methods include (i) TD-MS, which can directly analyze intact proteoforms and preserve native structural information, and (ii) 2DE-MS, which enables high-throughput separation of proteoforms across a wide molecular weight range (Zhan et al. 2018a, 2018b, 2019). Integration of these techniques with structural biology approaches and computational tools facilitates the elucidation of proteoform-specific structures and functions (Zhan et al. 2024; Fang et al. 2025). These technological advances provide the foundation for translating proteoform insights into personalized therapeutic strategies.

### 7.1. Technical foundation

**Figure 5.**
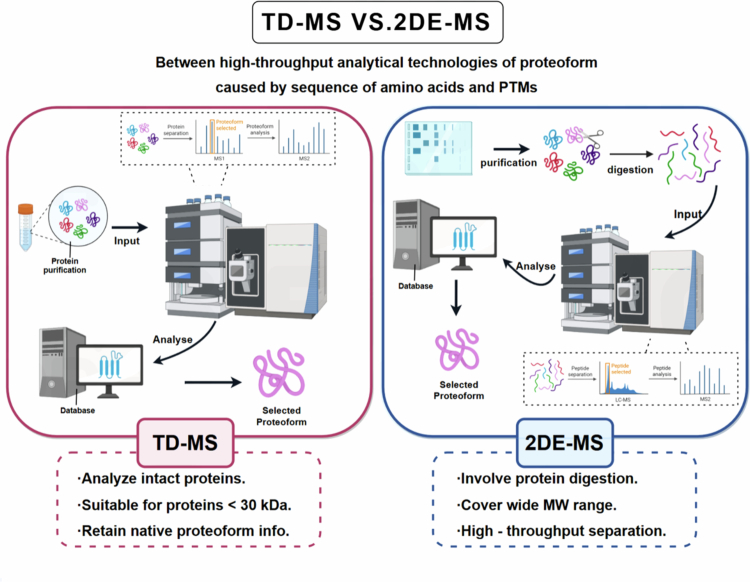
Comparison of high-throughput analytical technologies for identifying proteoforms derived from amino acid sequences and PTMs. This diagram illustrates two approaches: (a) TD-MS, where purified proteins are directly analyzed as intact molecules (suitable for proteins <30 kDa, retaining native proteoform information) via MS with database integration to identify target proteoforms; (b) 2DE-MS, where proteins are first separated by 2DE, then purified, digested into peptides, and analyzed via MS – this approach enables high-throughput separation across a wide molecular weight range, with database-assisted identification of selected proteoforms.

Proteoformics can large-scale detect, identify, and quantify the complex proteoforms in a proteome (Zhan et al. 2018a; Yang et al. 2023; Fang et al. 2025). The commonly used methods include: (i) TD-MS, whose general procedure is to purify therapeutic proteins or drug-targeted proteins, followed by introducing them into a mass spectrometer for random fragmentation to perform tandem mass spectrometry (MS/MS) analysis for identification of amino acid sequence and PTMs. TD-MS has low throughput and is currently typically applicable to proteins with a molecular weight of less than 30 kDa. (ii) 2DE-MS, which includes different approaches such as 2DE- matrix-assisted laser desorption/ionization (MALDI)-MS, and 2DE-electrospray ionization (ESI)-MS, whose common procedure involves separating proteins based on isoelectric point and relative molecular weight, followed by enzymatic digestion to form tryptic peptides. 2DE-MS offers ultra-high throughput for the analysis of human proteoforms (Zhan et al. 2018a, 2018b, 2019). In terms of structural analysis, MS is combined with structural biology techniques to study the structures of different proteoforms and their associations with functions (Zhan et al. 2024; Fang et al. 2025). Additionally, computational biological tools and bioinformatics are being rapidly developed to analyze the large-scale proteoformics data (Su et al. 2024; Fang et al. 2025) (Figure 5).

### 7.2. Application value

#### 7.2.1. In-depth elucidation of protein functions and disease mechanisms

Proteoformics can reveal the diversities and regulatory mechanisms of proteins, benefiting from a better understanding of the biological functions and roles of proteins towards more accurate personalized drug therapy and personalized medicine (Su et al. 2024).

#### 7.2.2. Facilitating personalized drug therapy

Proteoformics enables to furtherly develop personalized drug therapy, particularly personalized therapeutic protein drugs that target specific proteoforms, which will contribute to understanding disease mechanisms, identifying new signaling pathways and novel drug targets, providing scientific data and theoretical basis to develop novel drug, assisting clinical doctors in assessment of health risk, formulating more cost-effective strategies for targeted prevention, promoting the development of innovative technologies, and offering more convenient therapeutical strategies for individual patients, and ultimately benefit human society and affected individuals as a whole (Su et al. 2024; Fang et al. 2025).

#### 7.2.3. Promoting personalized medicine development

Proteoformics helps design customized therapeutic regimens targeting specific proteoforms, including optimization of drug dosage and selection of appropriate drug combinations. It even enables the customization of specific drugs for intervention against particular proteoforms. Furthermore, it provides new drug targets and technologies to accelerate the discovery and development of novel drugs, promotes the research and clinical practice of personalized medicine, and enhances therapeutic efficacy, and reduces side-effective reactions, which will highly promote the scientific research progress in the field of biomedicine (Su et al. 2024; Yang et al. 2025; Fang et al. 2025).

## 8. Disease proteoform biomarkers for TPfD in personalized medicine

Proteoforms, with their disease-specific expression and modification patterns, serve as key biomarkers for precision medicine (Aebersold et al. 2018; Fang et al. 2025). In cardiovascular diseases, TD-MS proteomics identified pathogenic phosphorylation states of proteins cardiac troponin I (cTnI) and tropomyosin 1.1 (Tpm1.1) in hypertrophic cardiomyopathy (HCM), providing early diagnostic markers and mechanistic insights (Tucholski et al. 2020). In neurodegenerative diseases, proteoforms of amyloid *β*, tau, *α*-synuclein, TDP-43, and huntingtin – modified by phosphorylation, acetylation, SUMOylation, and isomerization – regulate disease progression (Schaffert and Carter 2020). Multi-omics integration further expands this landscape: for example, combined ubiquitinomics and transcriptomics in sigmoid colon cancer identified deubiquitinases (DUBs) that stabilize pathogenic proteoforms, promoting tumor proliferation and metastasis (Yang et al. 2023). These examples underscore the value of proteoform biomarkers in disease stratification and therapeutic targeting. In the field of disease research, differences in proteoforms (including variations in amino acid sequences, and differences in PTMs, etc.) serve as the core biological basis for achieving precise disease typing, analyzing pathological mechanisms (e.g. abnormal activation of signaling pathways, and protein dysfunction), and conducting prognostic evaluation (Smith and Kelleher 2018; Li et al. 2022; Zhan et al. 2024).

In specific disease research, the application of proteoform biomarkers has made significant progress. In the field of cardiovascular diseases, the Tucholski team employed a top-down proteomics approach based on LC–MS and discovered pathogenic proteoforms with specific phosphorylation states in myocardial sarcomeric proteins, namely cardiac troponin I (cTnI) and tropomyosin 1.1 (Tpm1.1), in the myocardial tissue of patients with hypertrophic cardiomyopathy (HCM). These phosphorylation-modified protein variants directly contribute to the pathological disruption of myocardial cell contractile function, serving as pivotal molecular biomarkers for the early detection of HCM and investigations into its underlying mechanisms (Tucholski et al. 2020). In neurodegenerative diseases, many proteoforms act as regulators of such diseases. To illustrate: AD links to the phosphorylations of protein tau or amyloid *β*, alongside the formation of aspartic acid isomers in amyloid β; Parkinson’s disease (PD) correlates with *α*-synuclein modifications including 4-hydroxy-2-nonenal adduction, deacetylation, phosphorylation, and O-GlcNAcylation; amyotrophic lateral sclerosis (ALS) involves SUMOylation of superoxide dismutase, and 1acetylation and phosphorylation of transactive response DNA-binding protein 43; and Huntington’s disease (HD) ties to the phosphorylation in huntingtin protein (Schaffert and Carter 2020). Multi-omics integration analysis technologies (e.g. combined analysis of ubiquitinomics and phosphoproteomics) have further advanced the systematic analysis of proteoform regulatory networks. Hua Yang and colleagues successfully screened out members of the deubiquitinase (DUBs) family closely related to patient prognosis by integrating ubiquitination modification group data and transcriptome data of patients with sigmoid colon cancer, and clarified the downstream pathogenic proteoform substrates regulated by these DUBs. This study found that the high expression of specific DUBs can inhibit the ubiquitin-mediated degradation of target proteins, leading to the abnormal accumulation of pathogenic proteoforms in the tumor microenvironment, thereby promoting tumor proliferation and metastasis. This discovery provides a new direction for prognostic stratification and targeted therapy of sigmoid colon cancer (Yang et al. 2023) (Table 2).

**Table 2.** Disease proteoform biomarkers for TPfD in personalized medicine.

Disease type	Proteoform	Modification characteristic	Biological consequence	References
HCM	Cardiac sarcomeric protein cTnI	Specific phosphorylation state	Disrupts myocardial cell contractile function, contributing to pathology.	Tucholski et al. (2020)
Sigmoid colon cancer	Target proteins regulated by DUPs	Abnormal ubiquitination modification	Stabilization of oncogenic proteins, promoting tumor proliferation and metastasis.	Yang et al. (2023)
Alzheimer's disease (AD)	Tau protein	Hyperphosphorylation	Loss of microtubule-stabilizing function; aggregation into neurofibrillary tangles.	Parra Bravo et al. (2024)
Breast cancer	O-glycan	Glycosylation	Alters cell adhesion and signaling, influencing cancer development and metastasis.	Song et al. (2015)
Astroglial lesions	GFAP	Isoform differences, phosphorylation modification	Complicates detection and clinical interpretation as a biomarker for reactive astrogliosis.	Gogishvili et al. (2025)
Pituitary neuroendocrine tumors	GH Protein	Alternative splicing, phosphorylation, glycosylation, etc.	Generates variants with distinct functions.	Yang et al. (2023)
Chronic lymphocytic leukemia	SF3B1 Protein	Alternative splicing	Mutant proteoform aberrantly regulates RNA splicing, promoting cancer cell survival.	Rossi et al. (2011)

Note: This table summarizes the key information of proteoform biomarkers in different disease types. It lists seven common diseases, the corresponding proteoforms that play a role in each disease, the specific modification characteristics of these proteoforms, and the relevant reference sources. This information provides a clear reference for understanding the association between proteoforms and diseases, as well as the role of proteoform modifications in disease pathogenesis, and lays a foundation for the research and application of proteoform-based personalized diagnosis and treatment strategies.

The identification of disease-specific proteoform biomarkers provides a foundation for precision medicine, but translating these biomarkers into actionable therapeutic targets requires systematic integration of multi-omics and network information. Computational methods that predict the associations between drugs and diseases based on network embedding offer powerful tools for this purpose. For instance, NEDD was developed, which is a network embedding-based method; namely, it integrates disease‒disease similarities, drug‒drug similarities, and known drug‒disease associations to construct a heterogeneous network (Zhou et al. 2020). The NEDD uses meta-paths of different lengths to capture high-order proximities within the network and learn low-dimensional representation vectors for diseases and drugs, which are then used to predict novel associations with a random forest classifier (Zhou et al. 2020). This approach achieved superior performance in drug repositioning tasks, demonstrating the value of integrating heterogeneous network information for target prioritization. Applying such network-based computational methods to proteoform-specific data could enable systematic prioritization of pathogenic proteoforms as therapeutic targets and prediction of their potential indications, accelerating the development of TPfD-based precision therapies. Beyond network embedding approaches, matrix factorization-based methods have also proven effective for drug repurposing by integrating heterogeneous biological data. An indicator-regularized non-negative matrix factorization (IRNMF) framework was developed for COVID-19 drug repurposing, which incorporates the Karush–Kuhn–Tucker conditions and an indicator matrix to constrain the factorization process (Tang et al. 2021). IRNMF can integrate drug similarity, virus similarity, and known drug‒virus associations to achieve robust performance in predicting novel drug candidates, with an AUC of 0.8127 in a 5-fold cross-validation on a drug‒virus dataset comprising 210 drugs and 34 viruses. This method exemplifies how systematic computational approaches can identify candidate drugs for specific disease contexts, a principle that aligns conceptually with the TPfD framework. Extending such matrix factorization techniques to incorporate proteoform-specific data – such as proteoform–disease associations and proteoform–drug sensitivity profiles – could further enhance the prioritization of pathogenic proteoforms as therapeutic targets and facilitate the discovery of proteoform-selective degraders.

## 9. Design of TPfD drugs

As an innovative precision drug delivery modality for personalized medicine, TPfD agents prioritize disease-specific proteoform biomarkers as their central therapeutic objectives. We propose to design highly targeted molecular tools (PROTAC-like molecules) to achieve accurate recognition and degradation of pathogenic proteoforms, thereby reversing the disease process at the molecular level and achieving the therapeutic goal. Among them, the PROTAC-like (proteolysis-targeting chimera-like) design strategy serves as the core technical framework for TPfD drug development, mainly focusing on three key dimensions: ligand optimization, linker system, and E3 ligase selection. The synergistic effect of these three dimensions determines the degradation efficiency, selectivity, and clinical applicability of the drug.

### 9.1. Ligand optimization

PTM is a widespread covalent process that modulates protein function and activity within biochemical pathways. Such modifications arise via proteolytic cleavage or the addition of modifying moieties (e.g. methyl, phosphoryl, acetyl, or glycosyl groups) to amino acid residues in a single protein. Also, certain PTMs exhibit crosstalk capabilities, which reshape protein structure and function to produce novel protein variants. Disruptions to an organism’s homeostatic balance are a defining feature of cancer (Pienkowski et al. 2023). The conformational traits and PTM-selective binding capacity of PROTAC-like constructs depend primarily on refined targeting ligand design – specifically, the creation of ligands that can discriminate between proteoforms with closely analogous structures (e.g. wild-type vs. mutant proteins, normally vs. abnormally modified proteins). Existing chemical proteomics techniques can detect cysteine-reactive covalent ligands for undruggable proteins (Backus et al. 2016); additionally, multiple identified covalent E3 ligase ligands can be engineered into PROTACs that degrade target proteins of interest (POI) (Ward et al. 2019; Isobe et al. 2020). The design of ligands capable of distinguishing proteoforms has been exemplified by recent advances. For example, the single-chain variable fragment (scFv) used in the SOD1-targeting BioPROTAC was derived from an antibody that specifically recognizes the misfolded conformation of SOD1, demonstrating that protein-based binders can achieve high selectivity for disease-associated conformers (Chisholm et al. 2025). In another case, the NUP98:KDM5A fusion protein contains SPOP degron motifs that enable its recognition by the SPOP E3 ligase, which was exploited to design a SPOP-bioPROTAC that selectively degrades the fusion oncoprotein while sparing wild-type NUP98 (Kirkiz et al. 2025). For small molecules, the covalent KRASG12C inhibitor MRTX849 was modified into PROTAC LC-2, which retains selectivity for the G12C mutant over wild-type KRAS, showing that structure-guided warhead optimization can yield proteoform-specific ligands (Bond et al. 2020). Pathogenic proteoforms usually exhibit unique epitope exposure (e.g. exposure of hidden domains) or spatial conformational changes due to variations in amino acid sequences or alterations in PTMs, which provide a molecular basis for the specific binding of ligands (Hassan et al. 2024). Meanwhile, resolving the three-dimensional structure of pathogenic proteoforms through structural biology (e.g. cryo-electron microscopy and X-ray crystallography) can guide the rational design of ligands: enabling the targeting end of PROTAC-like molecules to accurately bind to the specific conformational sites of pathogenic proteoforms while avoiding non-specific binding to normal proteoforms, thereby ensuring the targeting of the drug and reducing interference with normal physiological functions. This strategy is already successfully used in therapeutic research through targeting AR:Bavdegalutamide complex, a protein degrader based on PROTAC, which incorporates a cyclohexyl group to interact with the ligand-binding domain of AR, while a brain-enriched E3 ubiquitin ligase component is engaged to mediate polyubiquitination in AR. This agent drives efficient degradation of AR in prostate cancer cell lines (LNCaP and VCaP), with DC50 around 1 nM. A total of ~4000 proteins were detected in VCaP cells, which reveals that the use of 10 nM Bavdegalutamide for 8-h treatment caused selective depletion of AR with a maximum degradation efficiency (Dmax) up to 85%. Beyond wild-type AR, Bavdegalutamide also targeted clinically actionable AR mutants (M896V, F877L, H875Y, and T878A) in preclinical models (Chirnomas et al. 2023). ACBI3’s ligand design draws on non-covalent KRAS-binding agents. Through structural resolution of the KRAS: ACBI3: VCB ternary complex (PDB ID 8QVU), the ligand’s binding interfaces with KRAS mutants (e.g. G12D, G12V) were refined. This allows ACBI3 to discriminate 13 commonly occurring KRAS oncogenic variants from wild-type KRAS, enabling pan-KRAS degradation.

### 9.2. Linker system

The core function of the linker system is to regulate the efficiency of the ternary complex. The length, rigidity, and chemical properties of the linker directly affect the stability and degree of spatial conformational alignment of the PROTAC-induced ternary complex (target proteoform-PROTAC-E3 ligase) (Troup et al. 2020; Chen et al. 2022; Dong et al. 2024). Optimizing the linker can significantly improve the degradation efficiency and selectivity for specific proteoforms. For example, the linker of ACBI3 uses a triazole structure instead of isoxazole and introduces a hydroxymethyl group to adjust the rigidity and length of the linker. This modification extends the half-life of the ternary complex (KRAS-ACBI3-VHL) to more than 2000 s and maintains the KRAS degradation effect in vivo beyond its pharmacokinetic existence time, demonstrating that linker optimization can strengthen the stability of the ternary complex (Tong et al. 2024). By adjusting the length of the linker, PROTACs can more effectively bridge target proteins with conformational changes caused by mutations or modifications and E3 ligases (Deng et al. 2020). The development of responsive smart linkers (e.g. transformable linkers activated by MMP-2, highly expressed in the tumor microenvironment) further enhances tissue specificity and degradation precision (Dragovich 2022). The MMP-2-sensitive linker (containing the GPLGLAGC sequence) is cleaved by MMP-2 highly expressed in the tumor microenvironment, causing nanoparticles to transform from a spherical shape to nanofibers. This transformation prolongs the retention time of the drug in tumor tissues and improves the degradation efficiency of BRD4 and the effect of photodynamic therapy (PDT) (Tong et al. 2024). In the designed pH/cathepsin B sequentially responsive nanoparticles (PSRNs), PROTAC is combined with a polymer through a cathepsin B-sensitive tetrapeptide linker (GFLG), and the polymer contains a pH-sensitive monomer (EPA-MA). This linker system possesses a ‘sequential response’ characteristic: under the acidic tumor microenvironment conditions (pH 6.5–7.0), it first triggers the dissociation of nanoparticles into monomers smaller than 10 nm, promoting tumor penetration and cellular internalization; after entering the lysosome, the linker is cleaved by the highly expressed cathepsin B, releasing PROTAC. This design breaks through the single-response mode of traditional linkers and solves the problem of ineffective extracellular release of PROTAC through the sequential activation of ‘microenvironment response-intracellular enzyme response’ (Yang et al. 2024).

### 9.3. E3 ligase selection

Diseases exhibit tissue specificity, and for drugs to exert their effects, drug resistance needs to be overcome (Wei et al. 2025). The spatiotemporal expression profile and substrate preference of E3 ligases profoundly affect the degradation spectrum and therapeutic window of PROTACs. Since E3 ligases are responsible for recognizing substrates and their family size is much larger than that of E1 and E2, the TPfD strategy based on the UPS utilizes E3 ligases as targets for protein degradation (Deng et al. 2020). Literature indicates that around 12 distinct E3 ligases, including FEM1B, KEAP1, RNF114, DCAF11, DCAF16, DCAF15, AhR, MDM2, IAPs, VHL, CRBN, and RNF4, facilitate the degradation of target proteins of interest (POIs) via PROTACs (Zhang et al. 2021; Henning et al. 2022). The application of traditional CRBN/VHL is limited by tissue restrictions and acquired drug resistance (Zhong et al. 2024). Research has shifted towards exploring novel or tissue-enriched E3 ligases: (i) Tissue/disease-restricted E3 ligases: focusing on the design of ligands for tissue-specific E3 ligases could open key avenues for disease therapy. This approach may mitigate on-target and off-target toxicities linked to unintended target depletion. For example, bromodomain PROTACs that deplete BRD4 are associated with intestinal toxicity (Bolden et al. 2014) – prioritizing tissue-restricted ligases could address this issue. (ii) Drug resistance-overcoming E3 ligases: DCAF1-based PROTACs (e.g. DBt-10) can effectively degrade BTK mutants in CRBN-deficient drug-resistant cell models, breaking through the drug resistance bottleneck mediated by traditional E3 ligases (Montoya et al. 2024). The E3 ligase receptor DCAF1 (a component of the CRL4DCAF1 complex) was explored. It is an essential gene in tumor cells (Demeter2 score indicates high tumor dependence) and can still function in CRBN-deficient drug-resistant cells. The developed DCAF1-based PROTAC (e.g. DBt-10) can effectively degrade BTK mutants in CRBN-deficient BTK-resistant cells, with an IC50 as low as 0.137 μM (Schröder et al. 2024). (iii) In addition, the exploration of non-classical E3 ligases (e.g. DCAF15, RNF114) provides new options for targeting unique proteoforms in hematological malignancies and solid tumors (Nguyen and Busino 2020). Beyond their application in targeted protein degradation, E3 ligases themselves play critical roles in regulating disease pathways through ubiquitination of key signaling molecules. Recent studies have highlighted how modulating E3 ligase activity can influence disease progression. For example, a study demonstrated that the natural compound Isoorientin can reduce KDM4A levels to alleviate macrophage pyroptosis and atherogenesis, which in turn promotes SKP1-Cullin1-F-box (SCF) E3 ligase-mediated ubiquitination in NLRP3 (Wang et al. 2025). This ubiquitination leads to the degradation of NLRP3, thereby suppressing inflammasome assembly and subsequent inflammatory responses. This study illustrates how enhancing specific E3 ligase activity can have therapeutic benefits in inflammatory diseases such as atherosclerosis.

In the context of cancer, E3 ubiquitin ligases are being actively explored as therapeutic targets for cancer intervention. For example, DCAF-based PROTACs have therapeutic potential in lung cancer, which focus on DCAF13/15/16 isoforms as substrate receptors of the CRL4 E3 ubiquitin ligase complex (Hussain et al. 2025). These DCAF proteins mediate the ubiquitination and degradation of oncogenic drivers, including mutant KRAS, RBM39, and nuclear proteins, offering a strategy to overcome tumor heterogeneity and drug resistance. The development of DCAF-recruiting PROTACs exemplifies how exploiting endogenous E3 ligase pathways can be harnessed for precision oncology.

These examples underscore the dual role of E3 ligases in both physiology and therapy: they are not only tools for targeted protein degradation but also critical regulators of disease-relevant pathways. A deeper understanding of E3 ligase biology, including their tissue-specific expression, substrate specificity, and regulatory mechanisms, will inform the rational design of next-generation TPfD molecules and combination therapies.

### 9.4. Network-level considerations in TPfD design

Beyond the selection of individual E3 ligases and ligand optimization, a network-level understanding of target proteoforms and their interaction partners is crucial for effective TPfD design. Proteins do not function in isolation but as part of larger complexes and interaction networks. Pathogenic proteoforms may exert their effects by perturbing these networks, and targeted degradation of a single proteoform could have broader consequences on complex integrity and cellular function.

Computational methods for protein complex detection in PPI networks offer valuable tools for this network-level analysis. For example, the DPCMNE framework was developed to employ multi-level network embedding to capture global and local topological features in PPI networks, enabling more accurate prediction of protein complexes (Meng et al. 2022). By integrating such computational approaches with proteoform-specific data, it becomes possible to map the interaction landscape of disease-associated proteoforms and identify vulnerabilities that can be exploited by TPfD strategies. For example, understanding whether a pathogenic proteoform exists as part of a larger complex could inform the choice of E3 ligase (e.g. based on tissue-specific expression of ligases within that complex) or guide linker design to ensure proper ternary complex formation. Future TPfD development should increasingly incorporate network-level analyses to enhance specificity and efficacy.

## 10. Optimization of therapeutic regimens based on TPfD

Personalized TPfD therapeutic regimens are not fixed; they need to be dynamically optimized based on changes in the patient's proteoforms during treatment, and effective detection methods are crucial for achieving this goal.

During the treatment process, regular detection of proteoforms in the patient's body fluids (e.g. blood, cerebrospinal fluid) or tissue samples can real-time monitor the expression level and modification status of target protein proteoforms, as well as the degradation effect of the drug on them. For example, when using PROTAC-like drug molecules to treat tumors, the concentration changes of tumor-related proteoforms in the patient's serum are detected to determine whether the drug effectively degrades the target protein and whether new mutant proteoforms appear (Deng et al. 2020). If a decrease in the degradation efficiency of the target protein proteoform is found, it may be due to the emergence of proteoforms related to drug resistance. In this case, the therapeutic regimen needs to be adjusted in a timely manner, such as replacing the PROTAC-like molecule or combining it with other therapeutic methods. Within a TNBC (triple-negative breast cancer) xenograft mouse model, MDM2 expression in tumor tissues was quantified via Western blotting and immunohistochemistry; For instance, MDM2 protein levels were reduced by 44% at 72 hours post-treatment. The degradation efficacy of MDM2-PROTAC was assessed by integrating apoptotic markers, including Caspase-3 activity and Annexin-V positivity rate. It is important to note that these measurements reflect total MDM2 protein levels and do not distinguish between its various proteoforms. MDM2 is known to undergo multiple PTMs such as ubiquitination and phosphorylation, which can alter its stability, subcellular localization, and oncogenic functions (Zafar et al. 2023). Future studies employing proteoform-specific detection methods are needed to determine whether PROTAC-mediated degradation equally affects all MDM2 proteoforms or selectively targets pathogenic variants. Such information would enable more precise therapeutic optimization and align with the TPfD concept. If a decrease in MDM2 degradation efficiency occurs (e.g. potential drug resistance after long-term treatment), the drug dose can be adjusted or combined with other therapies (Adams et al. 2023).

The development of detection technologies provides strong support for the dynamic optimization of therapeutic regimens. Currently, MS technologies with high sensitivity, resolution, and throughput have served as a key tool for proteoform detection. For example, 2DE-liquid chromatography-MS/MS (2DE-LC/MS) enables comprehensive, precise identification and quantification of proteoforms in complex biological samples. In addition, technologies such as antibody microarrays and protein microarrays can also be used for rapid detection of specific proteoforms, providing timely detection results for clinical treatment. Based on the dynamic change data of proteoforms obtained from detection, the therapeutic regimen is dynamically optimized. If a certain PROTAC has a good degradation effect on the target protein proteoform and no obvious drug resistance-related proteoforms appear, this regimen can be continued; if drug resistance-related proteoforms emerge, it is necessary to redesign or screen PROTAC-like molecules targeting the new proteoforms from the database (Münick et al. 2024), or adjust the drug dose and combined medication methods. In a TNBC nude mouse xenograft model, the degradation of PRMT5 in tumor tissues was detected by Western blot and immunohistochemistry (the PRMT5 expression in the YZ-836P group decreased by 65%). In conjunction with clinical markers, including aspartate transaminase (AST), serum alanine transaminase (ALT), and creatinine (which showed no notable deviations), both treatment efficacy and safety were assessed. However, these assays measured total PRMT5 protein and did not differentiate among its proteoforms. PRMT5 exists in multiple splice variants and is subject to post-translational modifications such as methylation and phosphorylation, which can modulate its methyltransferase activity and interaction with binding partners (Zafar et al. 2023). Therefore, the observed 65% reduction in total PRMT5 may mask differential effects on functionally distinct proteoforms. Future TPfD-oriented studies should aim to develop degraders that selectively target pathogenic PRMT5 proteoforms and monitor their levels with proteoform-specific tools. If a decrease in PRMT5 degradation efficiency occurs (e.g. after long-term treatment), the dose can be adjusted or combined with MDM2 downstream pathway inhibitors (e.g. KLF5 inhibitors) (Kim et al. 2022). At the same time, combined with the patient's clinical symptoms, physiological indicators, and other information, the therapeutic effect is comprehensively evaluated to ensure the effectiveness and safety of the therapeutic regimen.

**Figure 6.**
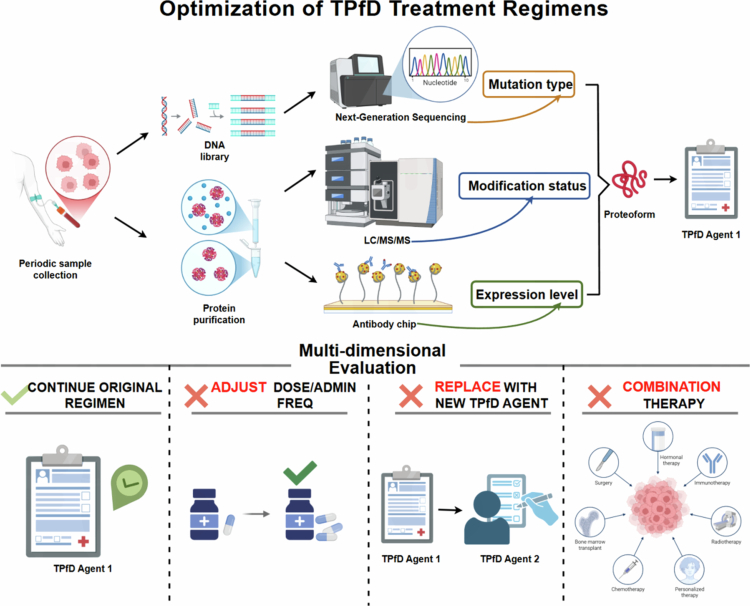
The optimization of TPfD regimens via multi-dimensional proteoform profiling. This flowchart outlines TPfD regimen optimization using multi-dimensional proteoform characterization (mutation type, modification status, expression level). Steps: periodic biological sample collection; parallel analyses: (1) DNA library + NGS for mutation type; (2) protein purification + LC/MS/MS for modification status; (3) antibody chip for expression level quantification. Data integration defines proteoform traits to guide therapeutic decisions, with four strategies: continue the original regimen, adjust dosage/frequency, switch to a new TPfD agent, or use combination therapy.

In summary, through the close combination of dynamic optimization of therapeutic regimens and detection, the precision and efficiency of personalized TPfD treatment can be achieved, maximizing the therapeutic effect, reducing the occurrence of adverse reactions, and bringing better treatment prognosis to patients (Figure 6).

## 11. Significance of TPfD

Due to the in-depth understanding of proteome functions, the scope of TPD has undergone significant changes. By centering on distinct proteoforms as key therapeutic targets, TPfD carries exceptional value, which manifests primarily in five key dimensions:

(i) Boosting treatment effectiveness: The core of disease typing is to identify pathological differences between individuals. By targeting specific proteoforms directly associated with diseases, TPfD enables therapeutic regimens to truly match the pathological characteristics of patients, thereby improving drug efficacy and minimizing exposure to ineffective drugs.
(ii) Minimizing side effects: During the action process of traditional drugs, they may exert non-specific effects on proteins in normal cells in the body, thereby triggering a series of side effects. TPfD has high specificity and only degrades abnormal proteoforms related to diseases, with minimal impact on proteins in normal cells (Su et al. 2024).
(iii) Expanding the range of treatable diseases: In traditional drug treatment, the treatment methods for some diseases are very limited due to the lack of clear and actionable targets. Through in-depth research on proteomics, more potential proteoform targets related to diseases can be discovered, thereby providing new therapeutic ideas and methods for diseases that were previously difficult to treat.
(iv) Promoting innovation in drug research and development models: The design process of TPfD drugs integrates multi-disciplinary technical means such as proteoformics (Fang et al. 2025), structural biology, and medicinal chemistry, breaking the traditional drug research and development model dominated by a single discipline and promoting the formation of a new multi-disciplinary integrated drug research and development model.
(v) Enhancing the effective allocation of medical resources: In the traditional medical model, due to the lack of accurate disease typing and effective therapeutic regimens, some patients may undergo multiple treatment attempts, which not only delays the disease but also causes waste of medical resources. By improving therapeutic efficacy and reducing ineffective treatment, TPfD can enable medical resources to be more accurately allocated to patients in need, improving the utilization efficiency of medical resources.

The realization of TPfD's transformative potential is not solely a scientific endeavor but also depends on the broader ecosystem in which drug development and precision medicine operate. Recent analyses of the medical technology sector highlight the importance of spatial and organizational networks in shaping innovation capacity and healthcare outcomes. The network structure of the specialized, refined, distinctive, and innovative (SRDI) medical device of China is measured to firm based on parent-subsidiary contacts, revealing significant regional disparities in network connectivity and the concentration of core hubs in eastern coastal cities (Hu et al. 2025). Their findings demonstrate that factors such as economic development, openness to trade, and technological innovation are key determinants of a city's centrality in the medical technology network. For TPfD to achieve broad clinical impact, it must be supported by policies that foster cross-regional collaboration, strengthen innovation ecosystems in underserved areas, and facilitate the flow of knowledge, talent, and resources. Integrating TPfD development with strategic health policy planning will be essential to ensure that these advanced therapies contribute to reducing health disparities rather than exacerbating them. Future research should explore how emerging technologies can be aligned with regional development strategies to maximize their societal benefit.

## 12. Key challenges and future perspectives for TPfD

Despite its transformative potential, the successful implementation of TPfD has several critical challenges, which must be discussed to advance from concept to clinical application. Below, we outline the primary limitations and propose corresponding strategies and future research directions.

### 12.1. Key challenges

(i) Technical difficulty in designing proteoform‑specific ligands: The most fundamental challenge lies in developing ligands that can discriminate between closely related proteoforms, such as those differing by a single PTM, a point mutation, or an alternative splice variant. These subtle structural differences often involve small, dynamic, or conformationally altered surfaces that are difficult to target with conventional small‑molecule or biologic binders. Without precise discrimination, TPfD risks either sparing the pathogenic proteoform (inefficacy) or inadvertently degrading functionally essential variants (toxicity). This precision deficit has been a major contributor to the failure of many TPfD candidates in preclinical development.
(ii) Limited repertoire and tissue distribution of usable E3 ligases: The efficacy of TPfD hinges on recruiting an E3 ubiquitin ligase to the target proteoform. Currently, only a handful of E3 ligases (e.g. CRBN, VHL) are routinely used in PROTAC designs, and their expression varies across tissues, potentially limiting tissue-specific degradation and increasing off-target toxicity (Liu and Ciulli 2023).
(iii) Risk of off‑target degradation and unintended immune responses: Even a highly selective ligand may cross‑react with homologous proteoforms or unrelated proteins, leading to unintended degradation and potential toxicity. Moreover, TPfD agents, particularly those based on antibodies or protein domains, may elicit immunogenicity.
(iv) Pharmacokinetic and tissue‑penetration challenges: TPfD agents, especially those incorporating protein binders or large PROTACs, often exhibit suboptimal pharmacokinetics – short half‑life, poor bioavailability, and limited ability to penetrate solid tumors or cross biological barriers such as the blood–brain barrier.

### 12.2. Future perspectives

(i) Advancing proteoform-specific ligand discovery: The identification of ligands capable of distinguishing closely related proteoforms will require integration of multiple cutting-edge approaches. First, high-resolution structural techniques (e.g. NMR, X-ray crystallography, and cryo-EM) can decipher three-dimensional conformations of pathogenic proteoforms, revealing unique epitopes or binding pockets that can be selectively targeted (Banari et al. 2025). Second, chemoproteogenomics offers a powerful platform for proteome-wide profiling of reactive cysteines and other residues, enabling the discovery of variant-specific covalent handles (Desai et al. 2024). Third, artificial intelligence (AI) and machine learning can accelerate ligand design by predicting proteoform-specific binding interfaces and optimizing warhead selectivity. Future efforts should also explore DNA-encoded libraries (DELs) and phage display to screen for high-affinity, proteoform-selective peptides or antibodies.
(ii) Expanding the E3 ligase toolbox: Broadening the repertoire of usable E3 ligases is critical for achieving tissue-specific degradation and overcoming drug resistance. Recently, a study demonstrated the feasibility of developing a macrocyclic ligand targeting CUL3^KLHL20, validating it as a potent new E3 ligase system for degrading nuclear targets such as BET proteins (Farrell et al. 2022). Future research should prioritize: first, ligand discovery for tissue-enriched E3 ligases (e.g. MDM2, RNF4, DCAF family members) to enable cell-type-specific degradation; second, exploring non-canonical E3 ligases (e.g. KEAP1) that may offer unique substrate preferences (Wei et al. 2021); and third, developing conditional or switchable E3 ligase recruiters that are activated only in diseased tissues, thereby minimizing on-target off-tissue toxicity (Kamaraj et al. 2024).
(iii) Mitigating off-target effects and immunogenicity: To minimize unintended degradation and immune responses, several strategies can be pursued. Reversible or conditional PROTACs (e.g. photo-switchable, chemically inducible, or tumor-microenvironment-activated designs) can provide spatiotemporal control over degradation, reducing systemic exposure (Kamaraj et al. 2024). Proteome-wide selectivity profiling using mass spectrometry-based chemoproteomics should be integrated early in the development pipeline to identify potential off-targets. For protein-based TPfD agents (e.g. bioPROTACs), humanization and de-immunization strategies can reduce immunogenicity. Additionally, combination therapies with immune checkpoint inhibitors may help counteract any immunosuppressive effects.
(iv) Improving pharmacokinetics and tissue delivery: Determining proteoform-specific interactions to target drugs in a native cell environment (Lutomski et al. 2025). Enhancing the drug-like properties of TPfD agents is essential for in vivo efficacy. One promising approach is the conjugation of degraders to long-circulating carriers, such as the Fc domain of antibodies. A study demonstrated that Fc-conjugation increased the half-life of the small-molecule drug conjugate from ~0.5 to 28  h while maintaining target specificity, a strategy that could be adapted for PROTACs or TPfD molecules (Zheng et al. 2025). Drug delivery systems based on nanotechnology include exosomes, lipid nanoparticles, and polymeric nanoparticles, which offer opportunities to improve tumor accumulation, cross biological barriers, and reduce systemic toxicity (Zheng et al. 2025). Furthermore, prodrug strategies that are activated by tumor-specific enzymes (e.g. cathepsins, matrix metalloproteinases) can enhance tissue selectivity and minimize off-target effects.
(v) Integrating multi-omics and network biology: A deeper understanding of proteoform interaction networks will inform more rational TPfD design. Computational methods for protein complex detection in PPI networks, such as the DPCMNE framework can map the interaction landscape of disease-associated proteoforms and identify vulnerabilities that can be exploited by TPfD strategies (Meng et al. 2022). Integrating proteoformics data with transcriptomics, ubiquitinomics, and phosphoproteomics will enable systematic prioritization of pathogenic proteoforms as therapeutic targets and facilitate the discovery of proteoform-selective degraders.

## 13. Conclusion

Protein homeostasis is a fundamental pillar of cellular and organismal physiology, and its disruption underpins numerous major diseases, including metabolic disorders, tumors, and neurodegenerative diseases. Traditional therapeutic strategies rely on an occupancy-driven mechanism with limitations: poor selectivity, drug resistance, and ineffectiveness against undruggable targets lacking defined binding pockets. In contrast, TPD, inspired by cellular natural degradation systems, actively clears target proteins via mechanisms like the UPS, representing a paradigm shift in drug development.

Among TPD technologies, PROTACs have emerged as a leading force, with their bifunctional design enabling precise recruitment of target proteins and E3 ligases for inducing ubiquitination and degradation. PROTACs offer advantages such as event-driven activity, low-dose efficiency, and the capability of targeting previously undruggable proteins, with over 6000 molecules developed to date and several entering clinical trials. However, PROTACs-based TPD faces critical challenges, including poor tissue permeability due to large molecular weight, limited diversity of E3 ligases, and, most importantly, inability to distinguish functionally heterogeneous proteoforms, which undermines their precision and therapeutic efficacy.

The rise of proteoformics, a field focused on systematically analyzing the diversity, dynamics, and functional implications of proteoforms, provides a solution to this bottleneck. By leveraging technologies such as TD-MS and single-cell proteomics, proteoformics enables precise identification of disease-specific pathogenic proteoforms and characterization of E3 ligase proteoform diversity. This laid the foundation for the concept of TPfD, which shifts the focus from average protein populations to specific proteoforms.

TPfD achieves personalized precision therapy through three key steps: (i) associating disease subtypes with proteoform signatures to enable accurate molecular typing, as exemplified by EGFR proteoforms in lung cancer and tau proteoforms in AD; (ii) designing and screening personalized PROTACs tailored to specific proteoforms, thereby enhancing selectivity and reducing drug resistance; and (iii) dynamically optimizing treatment regimens based on real-time monitoring of proteoform changes using advanced detection technologies like MS.

In brief, TPfD represents a transformative approach in precision medicine, bridging proteoformics with TPD technology to overcome the drawbacks of traditional therapies and PROTACs. By enabling targeted degradation of pathogenic proteoforms, TPfD maximizes therapeutic efficacy while minimizing side effects, paving the way for personalized treatments that address the molecular heterogeneity underlying disease. Future advancements in proteoform characterization, PROTAC design, and dynamic therapeutic monitoring will further propel TPfD toward clinical translation, offering new hope for patients with diverse and complex diseases. Notably, recent studies on misfolded SOD1-targeting BioPROTACs (Chisholm et al. 2025), SPOP-based degradation of NUP98 fusion oncoproteins (Kirkiz et al. 2025), and KRASG12C-selective PROTACs (Bond et al. 2020) have provided experimental validation for the TPfD concept, paving the way for its further development toward clinical applications.

## Data Availability

All data and materials are provided in this article, which can be available publicly.
